# Dental and Medical Surgery

**Published:** 1849-04

**Authors:** 


### DENTAL AND MEDICAL SURGERY.

Dr. A. T——, intending to locate permanently in the town of ——, respectfully tenders his professional services to the citizens and public generally, WARRANTED. Having introduced into his practice a new and improved method of Filling Teeth, with Gold and other foils of three different kinds, by which double the amount of Dentistry is produced, and all irritation is removed from the Artery, or destroyed so as to give perfect ease to all those who have decayed teeth. Also, whole and half sets of carved work, with false Jaws and Gums, or with single or separated Teeth, (as may be preferred,) mounted on gold plate, and confined by atmospheric pressure sufficiently firm to sustain a weight of sixteen pounds, and will be furnished on better terms and quality than can be procured in the country.

Possessing superior skill and long experience in amputations and all other branches of his profession, he hopes to receive a liberal share of patronage. He will, when requested visit persons at their residences.

In tracing of the human mind,
 Through all its various courses,
Though strange, 'tis true, we often find
 It knows not its resources.
And men through life assume a part
 For which no talent they possess,
Yet wonder at, with all their art,
 They meet no better with success.
'Tis thus we see, through life's career,
 So few excel in their profession;
Whereas, would each man but appear
 In what's within his own possession,
We would not see such daily quacks
 (For quacks there are in every art)
Attempting, by their strange attacks,
 To ameliorate the mind and heart.
Let each man to his part assigned
 By profession, take his part to act,
And then few causes shall we find
 To call each man a quack.

Truth in Dentistry will prevail. Office in ——.*

* The above dental card we publish for the benefit of the curious and those who may imagine dentistry is not a progressive science. We would draw attention to the "warrant," the "three kinds of foil," besides the gold, the irritation of the artery, the "false jaws," the superior skill and long experience in amputations; but, above all, the exquisite sentiment so admirably expressed in the poetry.—[CIN. ED.

